# Lehrbuck Der Hebammenkunst

**Published:** 1890-04

**Authors:** 


**Lehrbuch der Hebammenkunst.** Von Dr. BERNHARD SIGMUND SCHULTZE, Geheim-Hofarth, öff. ord. Prof. der Geburtshülfe, Director der Entbindungsanstalt und der Hebammenschule zu Jena, Mitglied der Medical Commission des Grossherzogth, Sachsen. Leipzig: Verlag von Wilhelm Englemann. 1889; pp. 380.

The ninth edition of this popular work is now presented to the German midwives, and, judging from the reception given the former editions, this ninth will be as enthusiastically received.

The midwife plays a more important rôle in Germany than in America, and is considered a part of the medical profession. It is no wonder, then, that such excellent treatises as Schultze's should be written especially for them. The language is free from technicalities, the construction simple and interesting, the illustrations pointed and lucid. The book is divided into paragraphs, each one taking up a certain point and discussing it briefly, but sufficiently to convey to the midwife the idea of the author. The descriptions of the various phases, with instructions how to act, the duties and obligations of the midwife to the patient and physician, are forcibly expressed, and, if carried out, must make her an important factor in the obstetrical art.

The typographical work, including the execution of the wood-cuts —ninety-six in number—is of the first quality, and is a good specimen of the excellent work done at Leipzig. W. C. K.

